# HIPred: an integrative approach to predicting haploinsufficient genes

**DOI:** 10.1093/bioinformatics/btx028

**Published:** 2017-01-30

**Authors:** Hashem A Shihab, Mark F Rogers, Colin Campbell, Tom R Gaunt

**Affiliations:** 1 MRC Integrative Epidemiology Unit (IEU), University of Bristol, Bristol, UK; 2 Intelligent Systems Laboratory, University of Bristol, Bristol, UK

## Abstract

**Motivation:**

A major cause of autosomal dominant disease is haploinsufficiency, whereby a single copy of a gene is not sufficient to maintain the normal function of the gene. A large proportion of existing methods for predicting haploinsufficiency incorporate biological networks, e.g. protein-protein interaction networks that have recently been shown to introduce study bias. As a result, these methods tend to perform best on well-studied genes, but underperform on less studied genes. The advent of large genome sequencing consortia, such as the 1000 genomes project, NHLBI Exome Sequencing Project and the Exome Aggregation Consortium creates an urgent need for unbiased haploinsufficiency prediction methods.

**Results:**

Here, we describe a machine learning approach, called HIPred, that integrates genomic and evolutionary information from ENSEMBL, with functional annotations from the Encyclopaedia of DNA Elements consortium and the NIH Roadmap Epigenomics Project to predict haploinsufficiency, without the study bias described earlier. We benchmark HIPred using several datasets and show that our unbiased method performs as well as, and in most cases, outperforms existing biased algorithms.

**Availability and Implementation:**

HIPred scores for all gene identifiers are available at: https://github.com/HAShihab/HIPred.

**Supplementary information:**

Supplementary data are available at *Bioinformatics* online.

## Introduction

Technological advances and the falling costs of next-generation sequencing technologies have accelerated the identification of genetic variation in the human genome (The 1000 Genomes Project Consortium, 2012). The most common form of genetic variation is single nucleotide variants (SNVs) and small insertions/deletions (INDELs). Identifying which of these are functional promises to improve our understanding of the molecular mechanisms of human disease and lead to novel treatments. As a result, there is a plethora of *in silico* algorithms capable of predicting the functional impact of SNVs and INDELs, e.g. (Choi *et al.*, 2012; Kircher *et al.*, 2014; Ritchie *et al.*, 2014; Shihab *et al.*, 2013, 2015). On the other hand, loss-of-function (LoF) variants, i.e. truncating mutations and whole gene deletions, have traditionally been considered to be rare and highly deleterious. However, there is growing evidence to suggest that LoF variants are common amongst healthy individuals (MacArthur *et al.*, 2012; Ng *et al.*, 2008; Pelak *et al.*, 2010). Haploinsufficiency, whereby a single copy of a gene product is not sufficient to maintain the normal function of the gene, is just one possible biological mechanism implicating LoF variants to abnormal phenotypes (Veitia and Birchler, 2010). Prediction of haploinsufficiency is an important aspect to interpreting whole genome sequence data, in which each individual will have a number of nonsense and missense mutations. Therefore, accurate methods for identifying haploinsufficiency within the genome are of increasing importance.

A large proportion of the existing algorithms for predicting haploinsufficiency utilize biological networks, such as protein–protein interaction networks. However, it has been shown that commonly used biological networks are heavily affected by study bias (Steinberg *et al.*, 2015); i.e. well studied genes are over-represented with respect to the number of networks they are part of and the number of links they form within these networks. As a result, these methods tend to perform best on well-studied genes but underperform on less studied genes. Steinberg *et al.* (2015) constructed an unbiased genome-wide haploinsufficiency score (GHIS) by replacing these biological networks with co-expression networks. However, other potentially informative sources for functional annotation include the Encyclopaedia of DNA Elements (ENCODE) consortium (The ENCODE Project Consortium, 2012) and the NIH Roadmap Epigenomics Project (Roadmap Epigenomics Consortium *et al.*, 2015). Following our previous work (Shihab *et al.*, 2015), we describe a machine learning approach (called HIPred) that integrates genomic and evolutionary features with functional annotations from ENCODE and NIH Roadmap Epigenomics to predict haploinsufficiency. We observe improved performance when compared with five existing methods, but without the potential study bias described earlier. Pre-computed HIPred scores for all gene identifiers (GRCh37) are available at: https://github.com/HAShihab/HIPred.

## 2 Materials and methods

### 2.1 Datasets

We assembled two datasets for training: our positive dataset was constructed using 299 known haploinsufficient genes from (Dang *et al.*, 2008) and our negative dataset was constructed using 386 putative loss-of-function tolerant (LoFT) genes from (MacArthur *et al.*, 2012). After removing records with conflicting annotations and ambiguous mappings, we retained 298 haploinsufficient and 386 LoFT genes.

Following a similar procedure described in Steinberg *et al.* (2015), we used the following benchmarks from (Petrovski *et al.*, 2013) to evaluate the performance of HIPred: 175 genes listed as haploinsufficient in OMIM (OMIM HI), 108 genes listed as haploinsufficient with known de novo mutations in OMIM (OMIM HI *de novo*), 91 genes for which a heterozygous gene knockout causes ‘lethality’ phenotypes in mouse (MGI Lethality) and 95 genes for which a heterozygous gene knockout causes seizures in mouse (MGI Seizures). Next, we collected a list of 59 genes disrupted by *de novo* LoF mutations in autism probands (ASD1) (Iossifov *et al.*, 2012) and a further 64 genes disrupted by *de novo* LoF mutations in other sets of autism probands (ASD2). (Neale *et al.*, 2012; O’Roak *et al.*, 2012; Sanders *et al.*, 2012). The composition of haploinsufficient genes across these benchmarks, and their overlap with our training data, is summarized in Supplementary Table S1. The actual genes used in these benchmarks are given as Supplementary Material.

Finally, for each gene, we also obtained the number of associated publications in PubMed using the NCBI Entrez Search and Retrieval System and used this as a measure of how ‘well-studied’ these genes are.

### 2.2 Feature groups

Following our previous work (Shihab *et al.*, 2015), we annotated our datasets using a number of *feature groups*, which could be predictive of haploinsufficiency. A detailed description of these feature groups can be found in Supplementary Table S2, but a short description is as follows:

- *Genomic and evolutionary*: we used a number of genomic properties such as the length of the gene, number of transcripts and the average number of predicted protein domains across transcripts. A comprehensive set of conservation-based measures, such as dN/dS ratios between human and 65 different species (one-to-one orthologues), was also used. In addition, we also tested whether the number of observed rare variants (MAF < 0.01) from the Exome Aggregation Consortium (ExAC) (Lek *et al.*, 2016), the number of expected rare variants across the gene, and a *z*-score representing the deviation of observed from expected added any predictive value.
- *Histone modifications*: we used regions of enrichment based on histone ChIP-seq peak calls from ENCODE and NIH Roadmap Epigenomics.
- *Open chromatin*: we used regions of enrichment based on DNase-seq and Formaldehyde-Assisted Isolation of Regulatory Elements peak calls from ENCODE and NIH Roadmap Epigenomics.
- *Transcription factor-binding sites*: based on PeakSeq and SPP peak calls for 119 transcription factors across 77 cell lines from ENCODE.
- *Gene expression*: based on RNA-seq signal coverage using consolidated epigenomes from NIH Roadmap Epigenomics.
- *Methylation*: based on whole genome bisulphite sequencing from NIH Roadmap Epigenomics.
- *Digital genomic footprinting sites*: for transcription factor recognition sequences within DNase-hypersensitive sites using consolidated epigenomes from the NIH Roadmap Epigenomics Project.
- *Networks*: we used measures of centrality from cell-type specific interactome and tissue-specific co-expression networks.

As described in the Supplementary Material, the majority of our feature groups comprise multiple annotations across a gene. For example, there could be multiple ChIP-seq values across a given region (one value for each position). In these instances, we used the median value across the region. We also tested other summary measures, specifically the mean and maximum value across a region; however, these summaries did not yield any significant improvements in the overall performance of our method (data not shown).

### 2.3 Data integration

**Fig. 1:**
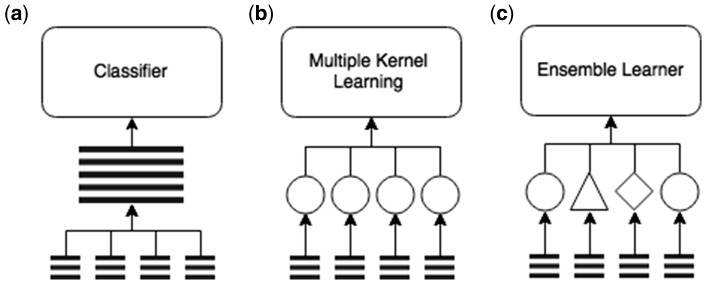
Methods for integrating feature groups: **(a)** feature groups are combined at the data level and fed into a single classifier; **(b)** feature groups are encoded as base kernels and combined using MKL; and **(c)** feature groups are used to construct heterogeneous base classifiers which are then combined using a stacking approach

The resulting product of our data preparation is several large matrices comprising data from the above feature groups, each of which can have different measurement scales. Therefore, we investigated three approaches for data integration (see Fig. 1). First, we evaluated data integration at the data level (i.e. concatenating datasets into a single matrix). This form of data integration is the simplest and most intuitive; however, combining feature groups in this way creates additional analytical challenges. For example, classifiers will need to handle a larger number of heterogeneous features. Therefore, we used a gradient boosted machine (Chen and Guestrin, 2016) as they can handle heterogeneous datasets, are robust to missing data and can estimate the relative importance of features. To illustrate the potential benefits of using a gradient boosted machine on this type of data, as opposed to alternative machine learning algorithms, we also evaluated the performance of a support vector machine (SVM) (Campbell and Ying, 2011) on the same task.

Next, we evaluated data integration based on multiple kernel learning (MKL). In MKL, feature groups are encoded into a corresponding base kernel $K_{\ell }$ (where ℓ=1,…,p if there are *p* feature groups), from which we can derive a composite kernel matrix $K=\sum_{\ell =1}^{p}\lambda_{\ell }K_{\ell }$. This composite kernel can then be used with a kernel-based classifier such as the SVM, which was the classifier used here. The $\lambda_{\ell }$ are kernel weights where $\sum_{\ell =1}^{p}\lambda_{\ell }=1$ and $\lambda_{\ell }\geq 0$. These weights can be adjusted according to the relative informative-ness of the different feature groups. We used an L1-norm to yield sparse solutions that implicitly excludes uninformative feature groups by assigning them zero weight.

Finally, we evaluated data integration based on stacking. Here, each feature group was tested against a number of machine learning algorithms, e.g. naïve Bayes, SVMs and random forests, and the best performing algorithm was chosen as the base classifier for the group $C_{\ell }$ (where ℓ=1,…,p if there are *p* feature groups). These base classifiers were then ’stacked’ (i.e. combined) using a logistic regression: $\text{log}\;\left(\frac{p}{1-p}\right)=\sum_{i=1}^{p}\beta_{i}C_{i}+\alpha$, where the β_*i*_ of each base classifier was deduced through the regression process. As with MKL, we used an L1-norm to implicitly exclude uninformative feature groups by assigning them zero coefficient.

We present our results using several performance statistics, such as the overall accuracy, sensitivity and specificity. In addition, we provide receiver operating characteristic (ROC) curves and area under the curve (AUC) statistics. Individual algorithm parameters, e.g. the SVM cost parameter C, were optimized through a 10-fold cross-validation and grid search.

To remove the potential bias caused by the random partitioning of the datasets during cross validation, we repeated our analysis 30 times and report the mean values and SDs above 0.01. In order to alleviate any performance artifacts arising from potential gene similarity within our training dataset, we performed a gene similarity analysis using NCBI’s BLASTCLUST algorithm using the following parameters: *-p F, -L 0.6, -b F* and *-S 10*.

Finally, we performed a feature selection analysis to identify important features and improve model interpretation. All analyses in this study were performed using scikit-learn (Pedregosa *et al.*, 2011), SHOGUN (Sonnenburg *et al.*, 2010) and xgboost (Chen and Guestrin, 2016).

### 2.4 Sequential learning

Both MKL and stacking rely on the assumption that data are available in every feature group for every training example. However, in practice, data can be absent from some feature groups, e.g. not every position in the genome will have annotations for Open Chromatin. We could use all available feature groups, but this would result in fewer training examples. With all values present for all feature groups, our training dataset consisted 156 haploinsufficient genes and just 52 LoFT genes. On the other hand, we could use a smaller number of feature groups, but with many more training examples. Therefore, we opted for an iterative sequential learning approach to determine the best combination of base kernels (MKL) and classifiers (stacking) (Rogers *et al.*, 2015). Here, we rank the base kernels/classifiers based on their individual cross-validation performance. Then, starting with the best performing base kernel/classifier, we iteratively add feature groups and retest the combined model using the same cross-validation procedure. If performance improves, then the feature group is added to the final model and the process is repeated until no more feature groups can be added.

### 2.5 Comparison with existing methods

For each of the benchmarks described in *Datasets*, we compared HIPred with 5 alternative methods that could be used to predict haploinsufficiency: predicted haploinsufficiency probabilities, HIS and HIS Imputed (Huang *et al.*, 2010); predicted gene indispensability scores (IS) (Khurana *et al.*, 2013); Residual Variance Intolerance Scores (RVIS) (Petrovski *et al.*, 2013); Evolutionary Intolerance (EvoTol) (Rackham *et al.*, 2014); and predicted genome-wide haploinsufficiency probabilities (GHIS) (Steinberg *et al.*, 2015). In addition, we evaluated the correlation between each of the methods evaluated based on the absolute Spearmando rank correlation coefficient.

## 3 Results

### 3.1 Performance of the method

On our training dataset, the performance of existing methods ranged from 0.6929 to 0.8549, with the HIS (Imputed) probabilities achieving the highest AUC (see Table 1). However, due to potential overlaps between this dataset and the datasets used to train these algorithms, the performances reported here may be over-inflated and may not represent the true generalizability of existing methods (see section 3.2 below).

**Table 1:** Performance of haploinsufficiency predictors on our training data

Method	Accuracy	Sensitivity	Specificity	Precision	NPV	AUC
EvoTol	0.6367	0.5577	0.7988	0.6905	0.6917	0.6929
GHIS	0.7069	0.7178	0.6327	0.6578	0.6951	0.7450
RVIS	0.8129	0.7895	0.7596	0.7059	0.8316	0.8329
HIS	0.6707	0.6683	0.8383	0.8354	0.6731	0.8412
IS	0.8478	0.8403	0.7017	0.6779	0.8547	0.8489
HIS (Imputed)	0.6195	0.5155	0.9257	0.8581	0.6867	0.8549
HIPred^a^	0.9032	0.8846	0.8919	0.8519	0.9167	0.8940

*Note:* NPV, negative predictive value; AUC, area under the curve.

a The reported performance of HIPred is the average performance observed across our repeated cross-validation procedure.

Next, we evaluated the performance of a gradient boosted machine, i.e. data integration at the data level. In terms of AUC, the performance of our gradient boosted machine outperformed all existing methods with an average AUC of 0.8940. Comparing the performance of a gradient boosted machine and SVMs, we achieved a nominal AUC of 0.8133 using SVMs; thereby highlighting the potential pitfalls of integrating large heterogeneous datasets at the data level.

In our experiments, the highest performing MKL model comprised seven feature groups and achieved an average AUC of 0.8747. Here, Genomic and Evolutionary was the highest performing individual feature group with an average AUC of 0.8179, followed by Open Chromatin and Histone Modifications from the NIH Roadmap Epigenomics Project (i.e. gappedPeak and narrowPeak) with an average AUC of 0.8103 and 0.8035, respectively. Histone Modifications from ENCODE yielded an average AUC of 0.7518. We observed a performance boost of 4.61% during the first stage of our sequential learning approach. However, we observed minor improvements at each subsequent iteration. Interestingly, MKL assigned the largest weight to Histone Modifications from ENCODE (0.6056), whose individual performance was ranked 4th overall, followed by Genomic and Evolutionary (0.2233) and Open Chromatin/Histone Modifications (narrowPeak) from NIH Roadmap Epigenomes Project (0.1701). A lower weight was assigned to gappedPeak and broadPeak (0.0008 and 0.0001, respectively), probably because of the similarities between these feature groups and the narrowPeak feature group (see Supplementary Tables S3 and S4).

The best performing stacked model comprised four feature groups and had an average AUC of 0.8866. As with MKL, Genomic and Evolutionary was the best performing feature group with an average AUC of 0.8196, followed by Open Chromatin and Histone Modifications (narrowPeak) with an AUC of 0.8794. However, in contrast to MKL, we observed a small performance boost of 0.38% after the initial sequential learning iteration. In our experiments, the most informative feature group was Genomic and Evolutionary with a regression coefficient of 4.0810, followed by Gene Expression and Open Chromatin (regression coefficients 2.1805 and 2.1391, respectively). Despite the best performing model comprising four feature groups during cross-validation, the final logistic regression assigned zero coefficients to one of these feature groups (see Supplementary Tables S5 and S6).

From our analysis, it would appear that all 3 data integration classifiers evaluated outperform existing methods, with a classifier based on integration at the data level performing best. However, the difference in performance between these three data integration techniques evaluated is marginal. Nonetheless, for maximum performance and improved model interpretation, the final version of HIPred is based on a gradient boosted machine. All subsequent analyses presented are based on this version.

Next, we tested for potential gene similarity (at the nucleotide level) within our training data using the NCBI BLASTCLUST algorithm. Using a minimum sequence identity and sequence coverage of 60%, we did not find any gene clusters.

**Fig. 2:**
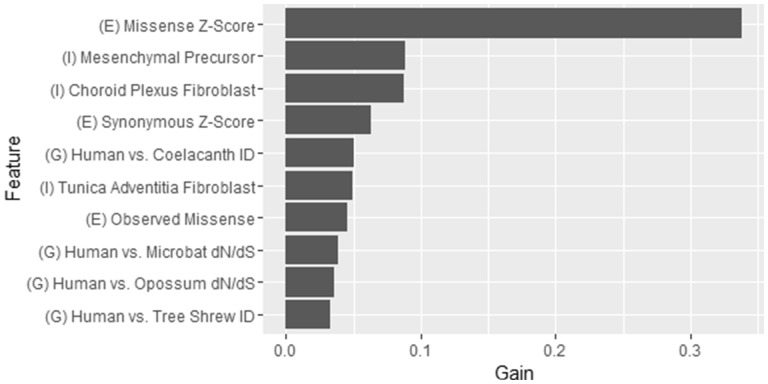
Informative features used for predicting haploinsufficient genes

Finally, we performed a feature selection analysis to identify the most informative features (see Fig. 2). The most important feature identified from this analysis was the ExAC (E) missense z-scores for the deviation of observed missense variants from expectation (gain = 0.34), followed by several cell-type specific interactomes (I), such as the Mesenchymal Precursor (gain = 0.09), and genomic/evolutionary features (G), such as the dN/dS ratios and percent identity with other Ensembl genomes. We also assessed the performance of our final model using a progressive number of features and found that a maximum tree depth of 2 yields optimal performance (see Supplementary Material).

### 3.2 Performance using known and candidate disease genes

We evaluated HIPred on a set of known human disease-associated genes and mouse model equivalents. After removing genes that were present in our training data (see Supplementary Table S1), we were left with 81 OMIM HI and 49 OMIM HI *de novo* genes. Following the procedure described in Steinberg *et al.* (2015), we matched these genes to an equal number of random genes based on gene length, which were assumed to be neutral. In general, HIPred outperformed the RVIS, EvoTol and GHIS across both OMIM datasets (see Table 2). Furthermore, HIPred marginally outperformed the HIS (both imputed and non-imputed) and Indispensability probabilities. However, these OMIM datasets comprise some of the most studied genes which could artificially inflate the observed performances of biased predictors. ROC curves are made available as Supplementary Figure S1.

**Table 2:** Performance of methods used for predicting haploinsufficiency on known disease genes and mouse models

Method	Accuracy	Sensitivity	Specificity	Precision	NPV	AUC
OMIM HI						
EvoTol	0.5232	0.5263	0.7358	0.7407	0.5200	0.6477
GHIS	0.8077	0.8630	0.3621	0.6300	0.6774	0.6845
RVIS	0.7593	0.8354	0.2807	0.6168	0.5517	0.6609
HIS	0.6604	0.7049	0.6923	0.7818	0.6000	0.7303
IS	0.7869	0.8354	0.5172	0.7021	0.6977	0.7451
HIS (Imputed)	0.4933	0.4722	0.8333	0.8095	0.5128	0.7156
HIPred	0.7606	0.7821	0.6026	0.6630	0.7344	0.7543
OMIM HI *de novo*						
EvoTol	0.5455	0.5455	0.7273	0.7273	0.5455	0.6959
GHIS	0.8361	0.8889	0.2973	0.6061	0.6875	0.7135
RVIS	0.8667	0.9149	0.2500	0.6143	0.6923	0.6965
HIS	0.7188	0.7568	0.6923	0.7778	0.6667	0.7599
IS	0.8286	0.8723	0.4857	0.6949	0.7391	0.7350
HIS (Imputed)	0.5455	0.5349	0.8333	0.8214	0.5556	0.7357
HIPred	0.8919	0.9130	0.5217	0.6562	0.8571	0.7902
MGI lethality						
EvoTol	0.4928	0.5000	0.7174	0.7292	0.4853	0.6258
GHIS	0.7576	0.8235	0.3958	0.6588	0.6129	0.6725
RVIS	0.6697	0.7600	0.3636	0.6706	0.4706	0.6523
HIS	0.5600	0.5926	0.7742	0.8205	0.5217	0.7210
IS	0.6949	0.7568	0.5200	0.7000	0.5909	0.7065
HIS (Imputed)	0.4676	0.4478	0.8537	0.8333	0.4861	0.7632
HIPred	0.7872	0.7973	0.7027	0.7284	0.7761	0.8143
MGI seizures						
EvoTol	0.5341	0.5287	0.7164	0.7077	0.5393	0.6611
GHIS	0.6748	0.7619	0.2879	0.5766	0.4872	0.5826
RVIS	0.7440	0.8222	0.2836	0.6066	0.5429	0.5748
HIS	0.4759	0.5000	0.6327	0.6786	0.4493	0.5428
IS	0.7000	0.7667	0.4143	0.6273	0.5800	0.5767
HIS (Imputed)	0.3854	0.3140	0.7231	0.6000	0.4434	0.5479
HIPred	0.7073	0.7333	0.5682	0.6346	0.6757	0.7024
ASD 1						
EvoTol	0.4016	0.2400	0.8478	0.6316	0.5065	0.4978
GHIS	0.7429	0.8085	0.3043	0.5429	0.6087	0.5185
RVIS	0.7468	0.8077	0.3778	0.6000	0.6296	0.6925
HIS	0.3563	0.2000	0.6316	0.3333	0.4615	0.4023
IS	0.5158	0.5660	0.4043	0.5172	0.4524	0.4621
HIS (Imputed)	0.3684	0.2174	0.7442	0.4762	0.4706	0.4426
HIPred	0.6049	0.6667	0.3542	0.5079	0.5152	0.4948
ASD 2						
EvoTol	0.4015	0.2931	0.7308	0.5484	0.4810	0.4428
GHIS	0.6757	0.7647	0.2245	0.5065	0.4783	0.5646
RVIS	0.6905	0.7593	0.3400	0.5541	0.5667	0.6259
HIS	0.4490	0.4130	0.7143	0.6552	0.4808	0.5609
IS	0.6275	0.6724	0.4630	0.5735	0.5682	0.5923
HIS (Imputed)	0.3750	0.2857	0.7273	0.5714	0.4444	0.5483
HIPred	0.6211	0.6667	0.4259	0.5373	0.5610	0.5640

*Note:* NPV, negative predictive value; AUC, area under the curve.

Next, we tested these methods using a set of genes for which a heterozygous gene knockout causes ‘lethality’ phenotypes and seizures in mouse. After removing genes that were also present in our training data, we were left with 75 MGI Lethality and 90 MGI Seizure genes, which were matched using the same procedure as above. From our analysis, it would appear that HIPred outperforms all other methods across these datasets. Although the performance of HIPred appears to drop on the MGI Seizures datasets, the drop in performance is not as drastic as that observed with other methods.

Using the number of associated publications in PubMed as a proxy of how well genes are studied, we tested whether the MGI dataset was enriched for less-studied genes compared with the OMIM datasets. Although the median number of publications was lower in the MGI datasets, we did not reach statistical significance using a Mann-Whitney U-test (*P =* 0.13 for MGI Seizures versus OMIM HI and *P =* 0.34 for MGI Lethality versus OMIM HI). Therefore, we also tested these methods on a set of candidate disease genes linked to autism (ASD1 and ASD2). These datasets were statistically enriched for less studied genes than the OMIM datasets (*P =* 0.02 for ASD1 versus OMIM HI and *P =* 0.01 ASD2 versus OMIM HI). After removing genes that were also present in our training data, we matched the remaining genes to to a random set of genes based on gene length as above. Our analysis shows that the performance of all methods drops significantly on these datasets, with RVIS performing best. The performance of HIPred is comparable to GHIS across the ASD datasets. However, it should be noted that we cannot be sure which ASD genes are casual (Steinberg *et al.*, 2015). Therefore, the results of this benchmark should be interpreted with some caution.

### 3.3 Rank correlation between methods

Following the above benchmarks, we tested the correlation in gene ranks between the methods (based on absolute Spearman’s rank correlation coefficient, see Table 3). Unsurprisingly, the highest correlation was observed between HIS and HIS (Imputed). Disregarding the HIS scores, rank correlations fall in the range 0.03–0.58, with correlations between EvoTol and all other methods being generally low. It appears that HIPred has a moderate correlation with all existing methods (coefficients range from 0.4994 to 0.5739, with the exception of EvoTol which yields a correlation coefficient of 0.0478).

**Table 3:** Spearman’s rank correlation between the methods

	RVIS	IS	EvoTol	HIS	HIS (imputed)	GHIS	HIPred
RVIS	1.0000						
IS	0.3293	1.0000					
EvoTol	0.0434	0.0675	1.0000				
HIS	0.3248	0.3534	0.0523	1.0000			
HIS (Imputed)	0.3512	0.3879	0.0609	0.9993	1.0000		
GHIS	0.5699	0.3783	0.0387	0.3598	0.3679	1.0000	
HIPred	0.4994	0.5250	0.0478	0.5652	0.5739	0.5031	1.0000

## 4 Discussion

In this study, we outlined HIPred, an integrative approach that combines genomic and evolutionary features with functional annotations from ENCODE and Roadmap Epigenomics to predict haploinsufficiency. We evaluated 3 approaches for data integration: integration at the data, kernel (MKL) and classifier (stacking) level; and observed improved performances over existing methods using all data integration techniques. In our experiments, we observed that MKL and stacking classifiers outperformed classifiers constructed for one type of data. However, we found that the most intuitive data integration technique, i.e. integration at the data level, outperformed other (more complex) data integration techniques. We observed comparable performances to existing methods using SVMs on the integrated data, but improved performances using a gradient boosted machine. The improved performance may be the result of the implicit feature selection performed in gradient boosted machines. Therefore, it may be possible to improve the performance of our MKL-based classifier using feature selection before data integration. However, our stacking classifier uses random forests (which are also tree-based methods similar to gradient boosted machines) for most feature groups and therefore performs some form of feature selection before data integration, so it is unclear how much benefit MKL would gain from feature selection. One main advantage to integration at the data level is the ability to capture the potential relationships between features *across* feature groups (which are missed using MKL and stacking based approaches).

We benchmarked HIPred using several datasets and have shown that our method performs as well as, and in most cases, outperforms existing algorithms. An important issue to consider when comparing the performance of any prediction algorithm is the benchmark being used. Here, it is preferable to use blind datasets, i.e. data that have not been used to train the algorithm, to minimize any bias in the observed performance. Although we took care to reduce this bias in our results by performing an extensive cross-validation analysis and excluding genes from our benchmarks that were also present in our training data, this level of testing is not possible with alternative methods as it would require training each method using common data. Therefore, the performance of alternative methods may be inflated. Furthermore, it has been shown that most biological networks used in existing methods are effected by study bias (Steinberg *et al.*, 2015), i.e. well-studied genes are over-represented in these networks compared with less studied genes. As a result, existing algorithms may not generalize well to less studied genes. For example, the performance of most existing algorithms drops when predicting on the MGI datasets, which comprise less studied genes. In contrast, HIPred doesn’t appear to be affected by this study bias and outperforms existing methods on these datasets.

Other important factors to consider when evaluating predictive methods are potential artifacts in performance arising due to gene similarity. Although we did not observe any gene similarities within our training data at the nucleotide sequence (up to 60% sequence similarity), we did not test for potential gene similarities at the protein sequence level.

The advent of large genome sequencing consortia, such as the 1000 genomes project (The 1000 Genomes Project Consortium, 2012), NHLBI Exome Sequencing Project (ESP) and the ExAC (Lek *et al.*, 2016), creates an urgent need for unbiased haploinsufficiency prediction methods such as HIPred.

## Supplementary Material

Supplementary Data
