# Supplementary material for: HIPred: an integrative approach to predicting haploinsufficient genes
Source: Bioinformatics. 2017 Jan 30;33(12):1751–7. doi: 10.1093/bioinformatics/btx028 (PMC5581952; doi:10.1093/bioinformatics/btx028)
Supplement: Supplementary Data [file btx028_supp.zip › Supplementary Data.doc]

Supplementary Data: HiPred - an integrative approach to predicting haploinsufficient genes

Hashem A. Shihab ^1*^, Mark F. Rogers ^2^, Colin Campbell ^2^, Tom R. Gaunt ^1*^

^1^ MRC Integrative Epidemiology Unit (IEU), University of Bristol, Bristol BS8 2BN, UK

^2^ Intelligent Systems Laboratory, University of Bristol, Bristol BS8 1UB, UK

^*^ To whom correspondence should be addressed: Hashem Shihab (H.Shihab@bris.ac.uk) or Tom R. Gaunt (Tom.Gaunt@bris.ac.uk)

**Supplementary Data**

**Feature Groups**

We annotated our datasets using the following *feature groups*:

A) *Genomic and Evolutionary*: the length of the gene, number of transcripts and predicted Pfam (Finn *et al.*, 2014) and SUPERFAMILY (Gough *et al.*, 2001) protein domains for each transcript was obtained from the BioMart community portal (Smedley *et al.*, 2015). For HIPred, we used the average number of protein domains across transcripts as opposed to raw counts. In addition, we used a comprehensive set of evolutionary-based measures, including sequence percent identity and dN/dS ratios from human to 65 different species (one-to-one orthologues) and to the highest scoring human paralog (obtained from Ensembl Compara (Vilella *et al.*, 2009)). We also used several conservation based measures, e.g. PhyloP (Pollard *et al.*, 2010) and PhastCons (Siepel *et al.*, 2005) scores, derived from the multiple sequence alignment of 46 and 100 vertebrate genomes to the human genome (Blanchette *et al.*, 2004).
B) *Histone Modifications*: we used ChIP-Seq peak calls for 14 histone modifications across 45 cell lines from ENCODE (The ENCODE Project Consortium, 2012) and narrow, broad and gapped regions of enrichment based on consolidated epigenomes from the NIH Roadmap project (Roadmap Epigenomics Consortium *et al.*, 2015).
C) *Open Chromatin*: we used DNase-Seq and Formaldehyde-Assisted Isolation of Regulatory Elements (FAIRE) peak calls across 119 cell lines from ENCODE and narrow, broad and gapped regions of enrichment based on consolidated epigenomes from the NIH Roadmap project.
D) *Transcription Factor Binding Sites*: based on PeakSeq and SPP peak calls for 119 transcription factors across 77 cell lines from ENCODE.
E) *Gene Expression*: based on RNA-seq signal coverage using consolidated epigenomes from the NIH Roadmap Epigenomics Project.
F) *Methylation*: based on whole genome bisulphite sequencing (WGBS) using consolidated epigenomes from the NIH Roadmap Epigenomics Project.
G) *Digital Genomic Footprinting Sites*: for transcription factor recognition sequences within DNase-hypersensitive sites using consolidated epigenomes from the NIH Roadmap Epigenomics Project.
H) Networks: we used measures of centrality from cell-type specific interactomes (Cornish *et al.*, 2015) and tissue specific co-expression networks (Pierson *et al.*, 2015).

The number of features within each of our datasets, along with their corresponding group, is presented in Supplementary Table 2. The majority of these features comprise multiple annotations across a specific region. For example, there are multiple PhyloP scores for a given genomic region (one for each base). In these instances, we took the median value across the region.

**Composition of Benchmarking Datasets**

The composition of haploinsufficient genes in our benchmarks is summarized in Supplementary Table 1. These genes were matched to random genes based on gene length, which were assumed to be neutral. The final benchmarks used in our analysis are available in Supplementary Data 2.

**Multiple Kernel Learning**

The best performing MKL model during cross-validation was achieved using a *C* parameter of 1000. The performance of constituent kernels, i.e. SVMs built on individual feature groups, is listed in Supplementary Table 3. Here, *Genomic and Evolutionary* was the best performing feature group with an average AUC of 0.8179, followed by *Open Chromatin and Histone Modifications* with an average AUC of 0.8103 (*gappedPeak*) and 0.8035 (*narrowPeak*).

The performance of our MKL-based classifier at each iteration of our sequential learning process is listed in Supplementary Table 4. Here, the process begins with the best performing constituent kernel and combines the next best kernel (with respect to AUC) via MKL. If the overall performance improves, then the two kernels are kept and the process iterates (i.e. starting with these kernels, iterate through the remaining kernels in descending order). The process is halted when all kernels have been combined and no improvement observed.

The average AUC of MKL is 0.8640 when the top two kernels are combined. This is a performance increase of 4.61% over the top performing base kernel. After this initial iteration, subsequent performance gains are marginal. The final MKL model comprises 7 feature groups and achieves an average AUC of 0.8747.

In contrast to expectation, the MKL weights (representing how informative a feature group is) do not follow the same order as the base kernel performance, i.e. the top performing base kernel does not receive the highest weight. The kernel with the highest weight is *Histone Modifications* from ENCODE (histone_macs). This kernel has an individual performance of 0.7518 and receives a weight of 0.6056. The other informative kernels are *Genomic and Evolutionaril*y with a kernel weight of 0.2233 and *Open Chromatin/Histone Modifications* (*narrowPeak*) with a kernel weight of 0.1701. Our MKL classifier also detects some additional discriminative power within the other kernels at later iterations of the sequential learning process and assigns them non-zero weights.

**Classifier Stacking**

The performance of constituent classifiers is listed in Supplementary Table 5. Here, *Genomic and Evolutionary* was the best performing feature group with an average AUC of 0.8756, followed by *Open Chromatin and Histone Modifications* (*narrowPeak* and *gappedPeak*) with an average AUC of 0.8196 and 0.8152, respectively.

The performance of a stacked classifier at each iteration of our sequential learning process is listed in Supplementary Table 6. As with MKL, the process begins the best performing constituent classifier and combined the next best classifier (with respect to AUC). If the overall performance improves, then the classifier is kept; otherwise, we move on to the next classifier. This process is repeated until all the constituent classifiers have been combined and no improvement observed.

The best performing feature group had an average AUC of 0.8756. When the top two performing base classifiers are stacked we achieved an average AUC of 0.8794. These marginal gains continue to be achieved throughout the sequential learning process. The final stacked model comprises 4 feature groups and achieves an average AUC of 0.8866.

Unlike MKL, the best performing base classifier receives the highest coefficient (representing how informative the feature group is). Interestingly, the 2^nd^ best performing base classifier (narrowPeak) receives a lower coefficient than the 3^rd^ best performing base classifier. Furthermore, while the stacked model performed well during cross-validation using 4 feature groups, the final regression assigned the 4^th^ feature group zero coefficient; effectively removing this group from the final model.

**Supplementary Table 1:** Overlap between haploinsufficient genes found in our training datasets and benchmarks.

|  | **Number of Haploinsufficient Genes** | **Overlap with Training** | **Final Number Used in Benchmark** |
| --- | --- | --- | --- |
| **Training** | 298 | - | - |
| **OMIM HI** | 175 | 94 | 81 |
| **OMIM HI de novo** | 108 | 59 | 49 |
| **MGI Lethality** | 91 | 16 | 75 |
| **MGI Seizures** | 95 | 5 | 90 |
| **ASD1** | 59 | 4 | 55 |
| **ASD2** | 64 | 3 | 61 |

**Supplementary Table 2.** The data and number of features used to represent our feature groups. Each dataset was partitioned according to its corresponding feature group and/or original source. For example, both *fdrPeaks* and *faire_fseq_peaks* represent open chromatin from ENCODE. In addition, NIH Roadmap provides peak calls based on DNase-Seq (*narrowPeak*, *broadPeak* and *gappedPeak*). Therefore, we use multiple datasets to represent open chromatin and allow our data integration techniques to choose which of these datasets are informative.

| **Name** | **Source** | **Group** | **# Features** |
| --- | --- | --- | --- |
| *genevo* | ENSEMBL, UCSC | A | 152 |
| *histone_macs* | ENCODE | B | 190 |
| *fdrPeaks* | ENCODE | C | 125 |
| *faire_fseq_peaks* | ENCODE | C | 24 |
| *narrowPeak* | NIH Roadmap | B, C | 1032 |
| *broadPeak* | NIH Roadmap | B, C | 979 |
| *gappedPeak* | NIH Roadmap | B, C | 979 |
| *peakSeq* | ENCODE | D | 495 |
| *spp* | ENCODE | D | 495 |
| *rna* | NIH Roadmap | E | 106 |
| *WGBS* | NIH Roadmap | F | 37 |
| *dgfootprints* | NIH Roadmap | G | 42 |
| *gnat* | Pierson *et al.* (2015) | H | 35 |
| *interactome* | Cornish *et al.* (2015) | H | 73 |

**Supplementary Table 3:** Performance of constituent SVM kernels.

| **Feature Group** | **AUC** |
| --- | --- |
| *genevo* | 0.8179 |
| *gappedPeak* | 0.8103 |
| *narrowPeak* | 0.8035 |
| *histone_macs* | 0.7518 |
| *broadPeak* | 0.7324 |
| *rna* | 0.6934 |
| *fdrPeaks* | 0.6860 |
| *spp* | 0.6900 |
| *WGBS* | 0.6787 |
| *peakSeq* | 0.6746 |
| *dgfootprints* | 0.6190 |
| *interactome* | 0.6166 |
| *faire_fseq_peaks* | 0.5965 |
| *gnat* | 0.5592 |

**Supplementary Table 4:** Performance of MKL via sequential learning

| **Iteration** | **Feature Groups** | **AUC** | **Weights** |
| --- | --- | --- | --- |
| 1 | *genevo* | 0.8179 | 1.0000 |
| 2 | *gappedPeak* | 0.8640 | 0.3204  0.6796 |
| 3 | *histone_macs* | 0.8649 | 0.2585  0.2191  0.5224 |
| 4 | *broadPeak* | 0.8652 | 0.2353  0.0964  0.5779  0.0905 |
| 5 | *narrowPeak* | 0.8665 | 0.2256  0.0039  0.5416  0.0811  0.1478 |
| 6 | *faire_fseq_peaks* | 0.8745 | 2.2326^-01^  8.9831^-04^  6.0563^-01^  1.2999^-04^  1.7007^-01^  1.0538^-05^ |
| 7 | *WGBS* | 0.8747 | 2.2331^-01^  8.3594^-04^  6.0557^-01^  1.1338^-04^  1.7008^-01^  9.5715^-06^  7.2769^-05^ |

**Supplementary Table 5:** Performance and type of base classifiers

| **Feature Group** | **Classifier** | **AUC** |
| --- | --- | --- |
| *genevo* | Random Forest | 0.8756 |
| *narrowPeak* | Random Forest | 0.8196 |
| *gappedPeak* | Random Forest | 0.8152 |
| *broadPeak* | Extra Trees | 0.8018 |
| *histone_macs* | Random Forest | 0.8011 |
| *rna* | Extra Trees | 0.7846 |
| *peakSeq* | Random Forest | 0.7549 |
| *spp* | Random Forest | 0.7452 |
| *fdrPeaks* | Random Forest | 0.7399 |
| *WGBS* | Extra Trees | 0.7219 |
| *dgfootprints* | Extra Trees | 0.7073 |
| *interactome* | Random Forest | 0.6613 |
| *faire_fseq_peaks* | Random Forest | 0.6394 |
| *gnat* | Extra Trees | 0.5771 |

**Supplementary Table 6:** Performance of stacking via sequential learning.

| **Iteration** | **Feature Groups** | **AUC (SD)** | **Weights** |
| --- | --- | --- | --- |
| 1 | *genevo.tab* | 0.8756 (0.0025) | 1.0000 |
| 2 | *narrowPeak.tab* | 0.8794 (0.0026) | 4.3945  3.1786 |
| 3 | *rna.tab* | 0.8847 (0.0024) | 4.1158  2.1554  2.6143 |
| 4 | *faire_fseq_peaks.tab* | 0.8866 (0.0021) | 4.0810  2.1391  2.1805  0.0000 |

**Supplementary Figure 1:** Performance of the methods evaluated on: A) list of haploinsufficienct genes from OMIM (OMIM HI); B) list of haploinsufficienct genes with *de novo* mutations from OMIM (OMIM HI *de novo*); C) human orthologs of one-to-one mouse knockouts causing lethality phenotypes (MGI Lethality); D) human orthologs of one-to-one mouse knockouts causing seizures (MGI Seizures); and E) / F) a set of genes disrupted by *de novo* LoF mutations in autism probands.

|  |  |
| --- | --- |
|  |  |
|  |  |

**References**

Blanchette,M. *et al.* (2004) Aligning Multiple Genomic Sequences With the Threaded Blockset Aligner. *Genome Res.*, **14**, 708–715.

Cornish,A.J. *et al.* (2015) Exploring the cellular basis of human disease through a large-scale mapping of deleterious genes to cell types. *Genome Med.*, **7**, 95.

Finn,R.D. *et al.* (2014) Pfam: the protein families database. *Nucleic Acids Res.*, **42**, D222–D230.

Gough,J. *et al.* (2001) Assignment of homology to genome sequences using a library of hidden Markov models that represent all proteins of known structure. *J. Mol. Biol.*, **313**, 903–919.

Pierson,E. *et al.* (2015) Sharing and Specificity of Co-expression Networks across 35 Human Tissues. *PLoS Comput Biol*, **11**, e1004220.

Pollard,K.S. *et al.* (2010) Detection of nonneutral substitution rates on mammalian phylogenies. *Genome Res.*, **20**, 110–121.

Roadmap Epigenomics Consortium *et al.* (2015) Integrative analysis of 111 reference human epigenomes. *Nature*, **518**, 317–330.

Siepel,A. *et al.* (2005) Evolutionarily conserved elements in vertebrate, insect, worm, and yeast genomes. *Genome Res.*, **15**, 1034–1050.

Smedley,D. *et al.* (2015) The BioMart community portal: an innovative alternative to large, centralized data repositories. *Nucleic Acids Res.*, **43**, W589–W598.

The ENCODE Project Consortium (2012) An integrated encyclopedia of DNA elements in the human genome. *Nature*, **489**, 57–74.

Vilella,A.J. *et al.* (2009) EnsemblCompara GeneTrees: Complete, duplication-aware phylogenetic trees in vertebrates. *Genome Res.*, **19**, 327–335.
