# Supplementary material for: HIPred: an integrative approach to predicting haploinsufficient genes
Source: Bioinformatics. 2017 Jan 30;33(12):1751–7. doi: 10.1093/bioinformatics/btx028 (PMC5581952; doi:10.1093/bioinformatics/btx028)
Supplement: Supplementary Data [file btx028_supp.zip › Supp_File_Superdelta.tar.gz!/S7_Theo_Proof.pdf]

1 Justification of the super-delta method

Model the ith expression level (denoted by y_{ij}^a) in a latent variable view as following. Within each phenotypic group ($a = A, B$), the (log)-expression level of the ith gene sampled from $j = 1, 2, \ldots, N_a$ arrays are realizations of $i.i.d.$ normal random variables with the following distribution

$$y_{ij}^a = \alpha + x_{ij}^a, \quad x_{ij}^a := \mu_i^a + \epsilon_{ij}^a, \quad \alpha \sim N(0, \eta^2), \quad \epsilon_{ij}^a \sim N(0, \sigma_i^2). \tag{1}$$

where α is a **slide-specific** factor, μ_i^a is the mean for each phenotype. $a = A, B$ represents different phenotypes. x_{ij}^a can be viewed as the "**oracle**" expression level, which has biological variation and possibly some other independent variation but no **slide-specific** noise. We are interested in testing the following hypotheses for each $i = 1, 2, ..., m$:

$$H_0^{(i)} : \mu_i^A = \mu_i^B, \qquad H_1^{(i)} : \mu_i^A \neq \mu_i^B. \tag{2}$$

Ideally, we would like to remove α from Model (1) by a normalization method, and then test hypotheses (2) based on two-sample t-statistics constructed from x_{ij}^a, the "**noise-free**" expressions:

$$t_i^* := \frac{\bar{x}_i^A - \bar{x}_i^B}{\hat{\sigma}_i^p \sqrt{\frac{1}{N_A} + \frac{1}{N_B}}}, \qquad \hat{\sigma}_i^p := \sqrt{\frac{\sum_{j=1}^{N_A} (x_{ij}^A - \bar{x}_i^A)^2 + \sum_{j'=1}^{N_B} (x_{ij'}^B - \bar{x}_i^B)^2}{N - 2}}. \tag{3}$$

Here $N = N_A + N_B$ is the total sample size. We will call these t_i^* as the **oracle** t-statistics henceforth. In practice, even the most effective normalization cannot perfectly recover x_{ij} from y_{ij}. Our previous studies show that commonly used normalization methods can effectively reduce sample variance explained in part by α, at a price of introducing certain bias to the normalized expressions, which in turn reduces statistical power and increases type I error rate.

In this study, we take a completely different approach. We will first compute pairwise differences called "**deltas**", defined as follows:

$$\delta_{ik,j}^a := y_{ij}^a - y_{kj}^a = x_{ij}^a - x_{kj}^a = \mu_i^a - \mu_k^a + \epsilon_{ij}^a - \epsilon_{kj}^a \sim N(\mu_i^a - \mu_k^a, \sigma_i^2 + \sigma_k^2). \tag{4}$$

Next, we compute two-sample t-statistics based on these deltas for all $i = 1, 2, \ldots, m$, $k = 1, 2, \ldots, m$, and $i \neq k$

$$t_{ik} := \frac{\bar{\delta}_{ik}^A - \bar{\delta}_{ik}^B}{s_{ik}^p \sqrt{\frac{1}{N_A} + \frac{1}{N_B}}}, \qquad s_{ik}^p := \sqrt{\frac{\sum_{j=1}^{N_A} (\delta_{ik,j}^A - \bar{\delta}_{ik}^A)^2 + \sum_{j'=1}^{N_B} (\delta_{ik,j'}^B - \bar{\delta}_{ik}^B)^2}{N - 2}}. \tag{5}$$

Let us denote the vector $(t_{i1}, t_{i2}, \ldots, t_{i,i-1}, t_{i,i+1}, \ldots, t_{im})$, by $\vec{t}_{i\cdot}$. Once we obtain $\vec{t}_{i\cdot}$, we can estimate the oracle statistic, t_i^*, by one of the following three approaches:

1. Sample mean. $\hat{t}_i^{(\text{mean})} := \frac{\sqrt{2}}{n} \sum_{k \neq i} t_{ik}$. The use of the multiplicative coefficient $\sqrt{2}$ will be justified later.
2. Sample median. $\hat{t}_i^{(\text{med})} := \sqrt{2}\text{Med}(\vec{t}_{i\cdot})$.
3. A robust estimator median fold trimmed median (MFTM), $\hat{t}_i^{(\text{robust})}$, defined as follows.

(a) Filter out a proportion (say $c = 20\%$) of the most *extreme* test statistics from $\vec{t_i}$. Here the most extreme values are determined by $|t_{ij}|$, the *absolute values* of t_{ij}. This step ensures that most, if not all, δ_{ij}'s normalized with DEGs will be excluded. Again we need to reiterate that filtering out 20% of t_{ij} according to $|t_{ij}|$ is not equivalent to filtering out 10% of smallest t_{ij} and 10% of largest t_{ij}. If up/down regulations are unbalanced, filtering according to $|t_{ij}|$ will automatically take care of it.

(b) Now estimate the center from the rest $1 - c$ of the δ_{ij}'s. We use the median (could also be the mean) for this purpose.

(c) In the end, we still need to multiply the trimmed median by $\sqrt{2}$.

1.1 The conditional interpretation of the estimation procedure

Because t_{ik} are computed from the difference of the ith gene expression and all the rest genes, they are *correlated* by construction. Besides, t_i^*, the oracle statistic, is not a population characteristic so the very "estimation" needs to be interpreted in an unconventional way. In this section, we will provide a rigorous interpretation based on conditional inference.

Proposition 1.1. *When conditioned on ϵ^a_{ij}, $\delta^a_{ik,j}$ and $\delta^a_{ik',j}$ are independent, for all $k \neq k'$ and $k, k' \neq i$.*

Proof. The proof is trivial because ϵ^a_{ij} are the randomness of the ith gene. Once it is fixed by conditioning, the only randomness in $\delta^a_{ik,j}$ is ϵ^a_{kj}, which are independent of each other. □

Corollary 1.2. *When conditioned on ϵ^a_{ij}, for any t_{ik} and $t_{ik'}$ in $\vec{t_{i\cdot}}$ such that $k \neq k'$, since they are functions of $\delta^a_{ik,j}$ and $\delta^a_{ik',j}$ respectively, these two-sample t-statistics are independent. The relationship can be seen more clearly from the following flow chart:*

$$t_{ik} \longleftarrow \delta^a_{ik,j} \longleftarrow \epsilon^a_{kj}, (\textit{given } \epsilon^a_{ij})$$

$$t_{ik'} \longleftarrow \delta^a_{ik',j} \longleftarrow \epsilon^a_{k'j}, (\textit{given } \epsilon^a_{ij})$$

Let us denote by $\mathcal{S}^0$ the set of all NDEGs (null genes) and $\mathcal{S}^1$ the set of all DEGs. Obviously, $d_k := \mu^A_k - \mu^B_k = 0$ for $k \in \mathcal{S}^0$ and $d_k \neq 0$ otherwise.
Based on simple calculation, we have the following conclusions.

Proposition 1.3. *When conditioned on ϵ^a_{ij}, sample mean difference of deltas, $\bar{\delta}^A_{ik} - \bar{\delta}^B_{ik}$, follows a normal distribution*

$$\bar{\delta}^A_{ik} - \bar{\delta}^B_{ik} \sim N\left(\underbrace{\mu^A_i - \mu^B_i}_{d_i} - (\underbrace{\mu^A_k - \mu^B_k}_{d_k}) + (\underbrace{\bar{\epsilon}^A_i - \bar{\epsilon}^B_i}_{\textit{cond. mean diff.}}),\ \sigma^2_k\left(\frac{1}{N_A} + \frac{1}{N_B}\right)\right). \tag{6}$$

Proof. By simple derivations, we can write $\bar{\delta}_{ik}^A$ in terms of $\bar{\epsilon}_i^A$ and $\bar{\epsilon}_k^A$:

$$\begin{aligned}\bar{\delta}_{ik}^A &= \frac{1}{N_A}\sum_{j=1}^{N_A}\delta_{ik,j}^A \\ &= \frac{1}{N_A}\sum_{j=1}^{N_A}(y_{ij}^A - y_{kj}^A) \\ &= \frac{1}{N_A}\sum_{j=1}^{N_A}(x_{ij}^A - x_{kj}^A) \\ &= \frac{1}{N_A}\sum_{j=1}^{N_A}[(\mu_i^A + \epsilon_{ij}^A) - (\mu_k^A + \epsilon_{kj}^A)] \\ &= \mu_i^A - \mu_k^A + \bar{\epsilon}_i^A - \bar{\epsilon}_k^A\end{aligned}$$

Similarly,

$$\bar{\delta}_{ik}^B = \mu_i^B - \mu_k^B + \bar{\epsilon}_i^B - \bar{\epsilon}_k^B$$

So we have the following (conditional) distributions:

$$\begin{aligned}\bar{\delta}_{ik}^A\Big|\epsilon_{ij}^A &\sim N(\mu_i^A - \mu_k^A, \frac{1}{N_A}\sigma_k^2) \\ \bar{\delta}_{ik}^B\Big|\epsilon_{ij}^B &\sim N(\mu_i^B - \mu_k^B, \frac{1}{N_B}\sigma_k^2) \\ \bar{\delta}_{ik}^A - \bar{\delta}_{ik}^B\Big|\epsilon_{i\cdot} &\sim N(d_i - d_k + (\bar{\epsilon}_i^A - \bar{\epsilon}_i^B), (\frac{1}{N_A} + \frac{1}{N_B})\sigma_k^2) \\ E\left(\bar{\delta}_{ik}^A - \bar{\delta}_{ik}^B\Big|\epsilon_{i\cdot}\right) &= d_i - d_k + (\bar{\epsilon}_i^A - \bar{\epsilon}_i^B) \\ &= \bar{x}_i^A - \bar{x}_i^B - d_k, \\ \mathrm{Var}\left(\bar{\delta}_{ik}^A - \bar{\delta}_{ik}^B\Big|\epsilon_{i\cdot}\right) &= \sigma_k^2\left(\frac{1}{N_A} + \frac{1}{N_B}\right).\end{aligned}$$

□

Proposition 1.4. *Sample variance of deltas computed in each phenotypic group, denoted by* $(s_{ik}^a)^2$, $a = A, B$, *has the following non-central* χ^2 *distribution conditional on* $\epsilon_{i\cdot}^a$.

$$\frac{(N_a - 1)(s_{ik}^a)^2}{\sigma_k^2} \sim \chi^2_{N_a-1;\lambda_{ik}^a}, \qquad \lambda_{ik}^a = \frac{(N_a - 1)(\hat{\sigma}_{\epsilon,i}^a)^2}{\sigma_k^2}. \tag{7}$$

Here $(\hat{\sigma}_{\epsilon,i}^a)^2 := \frac{1}{N_a-1}\sum_{j=1}^{N_a}(\epsilon_{ij} - \bar{\epsilon}_{i\cdot})^2$ *is the sample variance of* $\epsilon_{i\cdot}^a$.

Proof. By definition,

$$\begin{aligned}(s_{ik}^a)^2 &:= \frac{\sum_{j=1}^{N_a}\left(\delta_{ik,j}^a - \bar{\delta}_{ik}^a\right)^2}{N_a - 1} \\ &= \frac{\sum_{j=1}^{N_a}\left(\epsilon_{ij}^a - \bar{\epsilon}_i^a - \left(\epsilon_{kj}^a - \bar{\epsilon}_k^a\right)\right)^2}{N_a - 1} \\ &= \frac{1}{N_a - 1}\left(\epsilon_{i\cdot}^a - \epsilon_{k\cdot}^a\right)^T\left(I - \frac{1}{N_a}J\right)\left(\epsilon_{i\cdot}^a - \epsilon_{k\cdot}^a\right).\end{aligned}$$

Here J denotes an $N_a \times N_a$-dimensional matrix with all entries being 1. Simple calculation shows that $(I - \frac{1}{n}J)$ is symmetric, idempotent, with rank $N_a - 1$. In fact, it is the projection matrix onto $\text{span}(\mathbf{1})^\perp$, where $\mathbf{1}$ is a vector of all 1s. When conditioned on ϵ_{ij}^a, $\epsilon_{ij}^a - \epsilon_{kj}^a$ is a normal distribution with mean ϵ_{ij}^a and variance σ_k^2. Therefore

$$\frac{(N_a - 1)(s_{ik}^a)^2}{\sigma_k^2} \sim \chi^2_{N_a - 1; \lambda_{ik}^a}, \qquad \lambda_{ik}^a = (\epsilon_{i\cdot}^a)^T \left(I - \frac{1}{N_a} J \right) \epsilon_{i\cdot}^a = \frac{(N_a - 1)(\hat{\sigma}_{\epsilon,i}^a)^2}{\sigma_k^2}.$$

□

Corollary 1.5. *The pooled sample variance,* $(s_{ik}^p)^2 := \frac{(N_A-1)\left(s_{ik}^A\right)^2 + (N_B-1)\left(s_{ik}^B\right)^2}{N-2}$*, has the following conditional non-central* χ^2 *distribution representation*

$$\frac{N-2}{\sigma_k^2} \cdot (s_{ik}^p)^2 \sim \chi^2_{N-2; \lambda_{ik}}, \qquad \lambda_{ik} = \frac{(N-2)(\hat{\sigma}_{\epsilon,i}^p)^2}{\sigma_k^2}. \tag{8}$$

Here $(\hat{\sigma}_{\epsilon,i}^p)^2$ *is the sample pooled variance of* ϵ_i *computed from both phenotypic groups.*

Proof. This corollary is a direct consequence of Proposition 1.4 and the additivity of noncentral χ^2-distribution. □

Proposition 1.6. *The sample mean and sample variance of deltas are independent conditional on* $\epsilon_{i\cdot}$ *in each phenotypic group. Hence, the sample mean difference and sample pooled variance of deltas are independent.*

Proof. This is just because $\epsilon_{kj}^a - \bar{\epsilon}_k^a$ is independent of $\bar{\epsilon}_k^a$, for all j. □

Based on Proposition 1.3, Corollary 1.5, and Proposition 1.6, we have the following conclusion.

Lemma 1.7. t_{ik} *computed from deltas follows a* doubly non-central t*-distribution as follows*

$$t_{ik} \sim t''\left(N-2, \mu_{ik}, \lambda_{ik}\right), \quad \mu_{ik} := \frac{\bar{x}_i^A - \bar{x}_i^B - d_k}{\sigma_k \sqrt{\frac{1}{N_A} + \frac{1}{N_B}}}, \quad \lambda_{ik} := \frac{(N-2)(\hat{\sigma}_{\epsilon,i}^p)^2}{\sigma_k^2}. \tag{9}$$

Proof.

$$t_{ik} = \sqrt{N-2} \cdot \frac{\frac{\bar{\delta}_{ik}^A - \bar{\delta}_{ik}^B}{\sigma_k \sqrt{\frac{1}{N_A} + \frac{1}{N_B}}}}{\frac{\sqrt{N-2}}{\sigma_k} \cdot s_{ik}^p} \sim \sqrt{N-2} \cdot \frac{N\left(\mu_{ik}, 1\right)}{\chi_{N-2; \lambda_{ik}}}.$$

□

Lemma 1.8. *The conditional mean value of* t_{ik} *given* $\epsilon_{i\cdot}$*, which is a doubly noncentral* t*-distribution, has the following large-sample approximation*

$$\begin{aligned} E\left(t_{ik} | \epsilon_{i\cdot}\right) &= \mu_{ik} \sqrt{\frac{N-2}{2}} \frac{\Gamma\left(\frac{N-3}{2}\right)}{\Gamma\left(\frac{N-2}{2}\right)} \cdot H\left(\frac{1}{2}, \frac{N-2}{2}; -\frac{\lambda_{ik}}{2}\right) \\ &= \frac{\mu_{ik}}{\sqrt{1 + \frac{(\hat{\sigma}_{\epsilon,i}^p)^2}{\sigma_k^2}}} + O(N^{-1}) \\ &= \frac{\bar{x}_i^A - \bar{x}_i^B - d_k}{\sqrt{\sigma_k^2 + \hat{\sigma}_{\epsilon,i}^2} \cdot \sqrt{\frac{1}{N_A} + \frac{1}{N_B}}} + O(N^{-1}). \end{aligned} \tag{10}$$

In particular, for $k \in \mathcal{S}^0$,

$$E(t_{ik}|\epsilon_{i\cdot}) = \sqrt{\frac{1}{1+\frac{(\hat{\sigma}^p_{\epsilon,i})^2}{\sigma_k^2}}} \cdot t_i^* + O(N^{-1}). \tag{11}$$

for $k \in \mathcal{S}^1$,

$$\begin{aligned} E(t_{ik}|\epsilon_{i\cdot}) &= \sqrt{\frac{1}{1+\frac{(\hat{\sigma}^p_{\epsilon,i})^2}{\sigma_k^2}}} \cdot t_i^* + \frac{d_k}{\sqrt{\sigma_k^2+\hat{\sigma}^2_{\epsilon,i}} \cdot \sqrt{\frac{1}{N_A}+\frac{1}{N_B}}} + O(N^{-1}) \\ &= \sqrt{\frac{1}{1+\frac{(\hat{\sigma}^p_{\epsilon,i})^2}{\sigma_k^2}}} \cdot t_i^* + d_k \cdot O(N) + O(N^{-1}) = O(N). \end{aligned} \tag{12}$$

Proof. For simplicity, let us skip "$|\epsilon_{i\cdot}$" in the following derivation. Namely, all expectations/variance in the following proof are conditioned on $\epsilon_{i\cdot}$.

Let us denote $\frac{\bar{\delta}^A_{ik}-\bar{\delta}^B_{ik}}{\sigma_k\sqrt{\frac{1}{N_A}+\frac{1}{N_B}}}$ by U and $\frac{\sigma_k}{s^p_{ik}}$ by W, so $t_{ik} := U \cdot W$. By construction, we know that $U \sim N(\mu_{ik}, 1)$, $(N-2)W^{-2} = (N-2)\cdot\frac{(s^p_{ik})^2}{\sigma_k^2} \sim \chi^2_{N-2;\,\lambda_{ik}}$, and U, W are independent.

First, we will calculate some useful moments of W based on the large sample approximation.

Since $(s^p_{ik})^2$ follows a conditional scaled non-central χ^2-distribution, there exists a sequence of *i.i.d.* normal random variables $u_1, u_2, \ldots, u_{N-2}$ such that

$$W^{-2} := \frac{(s^p_{ik})^2}{\sigma_k^2} \overset{d}{=} \frac{\sum_{l=1}^{N-2} u_l^2}{N-2} = \overline{u^2}, \qquad u_l \sim N\left(\frac{\hat{\sigma}^p_{\epsilon,i}}{\sigma_k}, 1\right), \qquad u_l^2 \sim \chi^2_{1;\,\frac{(\hat{\sigma}^p_{\epsilon,i})^2}{\sigma_k^2}}.$$

$$Eu_l^2 = \frac{(\hat{\sigma}^p_{\epsilon,i})^2}{\sigma_k^2} + 1, \quad \mathrm{var}\left(u_l^2\right) = 2 + 4\cdot\frac{(\hat{\sigma}^p_{\epsilon,i})^2}{\sigma_k^2}, \quad \mathrm{SK}(u_l^2) = \frac{\sqrt{8}(1+3\cdot\frac{(\hat{\sigma}^p_{\epsilon,i})^2}{\sigma_k^2})}{(1+2\cdot\frac{(\hat{\sigma}^p_{\epsilon,i})^2}{\sigma_k^2})^{3/2}}.$$

Based on Taylor expansion, we have

$$\begin{aligned} EW := E\left(\overline{u^2}\right)^{-1/2} &= \left(Eu_l^2\right)^{-1/2} + \frac{3}{8}\cdot\left(Eu_l^2\right)^{-5/2}\cdot\frac{\mathrm{var}\left(u_l^2\right)}{N} + O(N^{-2}) \\ &= \frac{1}{\sqrt{1+\frac{(\hat{\sigma}^p_{\epsilon,i})^2}{\sigma_k^2}}} + \frac{3\left(1+2\frac{(\hat{\sigma}^p_{\epsilon,i})^2}{\sigma_k^2}\right)}{4\left(1+\frac{(\hat{\sigma}^p_{\epsilon,i})^2}{\sigma_k^2}\right)^{5/2}}\cdot N^{-1} + O(N^{-2}). \\ &= \frac{1}{\sqrt{1+\frac{(\hat{\sigma}^p_{\epsilon,i})^2}{\sigma_k^2}}} + O(N^{-1}). \end{aligned} \tag{13}$$

$$
\begin{aligned}
EW^2 &:= E\left(\overline{u^2}\right)^{-1} = \left(Eu_l^2\right)^{-1} - \frac{1}{2}\cdot\left(Eu_l^2\right)^{-2}\cdot\frac{\operatorname{var}\left(u_l^2\right)}{N} + O(N^{-2}) \\
&= \frac{1}{1+\frac{(\hat{\sigma}^p_{\epsilon,i})^2}{\sigma_k^2}} + O(N^{-1}).
\end{aligned} \tag{14}
$$

$$
EW^3 := E\left(\overline{u^2}\right)^{-3/2} = \left(1+\frac{(\hat{\sigma}^p_{\epsilon,i})^2}{\sigma_k^2}\right)^{-3/2} + O(N^{-1}). \tag{15}
$$

Therefore, based on the independence of the denominator and numerator,

$$
\begin{aligned}
E(t_{ik}|\epsilon_{i\cdot}) &= E\left(\frac{\bar{\delta}^A_{ik}-\bar{\delta}^B_{ik}}{\sigma_k\sqrt{\frac{1}{N_A}+\frac{1}{N_B}}}\right)\cdot E\left(\overline{u^2}\right)^{-1/2} \\
&= \frac{\mu_{ik}}{\sqrt{1+\frac{(\hat{\sigma}^p_{\epsilon,i})^2}{\sigma_k^2}}} + O(N^{-1}) \\
&= \frac{\bar{x}^A_i-\bar{x}^B_i-d_k}{\sqrt{\sigma_k^2+\hat{\sigma}^2_{\epsilon,i}}\cdot\sqrt{\frac{1}{N_A}+\frac{1}{N_B}}} + O(N^{-1}).
\end{aligned}
$$

As a special case, for $k \in \mathcal{S}^0$, $d_k = 0$, therefore

$$
E\left(t_{ik}|\epsilon_{i\cdot}\right) = \sqrt{\frac{1}{1+\frac{(\hat{\sigma}^p_{\epsilon,i})^2}{\sigma_k^2}}}\cdot t_i^* + O(N^{-1}).
$$

Similarly, we conclude that for $k \in \mathcal{S}^1$, $E\left(t_{ik}|\epsilon_{i\cdot}\right)$ is dominated by the $\dfrac{d_k}{\sqrt{\sigma_k^2+\hat{\sigma}^2_{\epsilon,i}}\cdot\sqrt{\frac{1}{N_A}+\frac{1}{N_B}}}$ term, which approaches ∞ with rate $O(N)$. □

Below we will prove a similar lemma for the median. But first, we will need to understand the asymptotic properties of the higher order moments of t_{ik}.

Proposition 1.9. *The conditional variance and skewness of t_{ik} have the following asymptotic representation*

$$
\operatorname{var}\left(t_{ik}|\epsilon_{i\cdot}\right) = 1 + \frac{(\hat{\sigma}^p_{\epsilon,i})^2}{\sigma_k^2} + O(N^{-1}), \tag{16}
$$

and

$$
\operatorname{SK}\left(t_{ik}|\epsilon_{i\cdot}\right) = O(N^{-1}). \tag{17}
$$

Proof. Let us use the same decomposition as in the previous proof, namely $t_{ik} := U\cdot W$.

For variance, we have

$$
\begin{aligned}
\operatorname{Var}(UW) &= E[(UW) - E(UW)]^2 \\
&= E\left(U^2W^2\right) - E^2(UW) \\
&= E\left(U^2\right)\cdot E\left(W^2\right) - (EU)^2\cdot(EW)^2 \\
&= \left(\operatorname{Var}(U) + (EU)^2\right)\cdot\left(\operatorname{Var}(W) + (EW)^2\right) - (EU)^2\cdot(EW)^2 \\
&= \left(\operatorname{Var}(U) + (EU)^2\right)\operatorname{Var}(W) + \operatorname{Var}(U)\,(EW)^2 + (EU)^2\cdot(EW)^2 - (EU)^2\cdot(EW)^2 \\
&= \left(\operatorname{Var}(U) + (EU)^2\right)\operatorname{Var}(W) + \operatorname{Var}(U)\,(EW)^2 \\
&= \frac{(1+\mu_{ik}^2)\sigma_W^2}{N} + 1 + \frac{(\hat{\sigma}^p_{\epsilon,i})^2}{\sigma_k^2} + O(N^{-1}) \\
&= 1 + \frac{(\hat{\sigma}^p_{\epsilon,i})^2}{\sigma_k^2} + O(N^{-1}).
\end{aligned}
$$

For skewness, we have the following computation. Denote $\frac{1}{\sqrt{1+\frac{(\hat{\sigma}^p_{\epsilon,i})^2}{\sigma_k^2}}}$ by ν_{ik},

$$
\begin{aligned}
E\left(UW - E(UW)\right)^3 &= E[(UW)^3 - 3\,(UW)^2\cdot E\,(UW) + 3\,(UW)\cdot E^2\,(UW) - E^3\,(UW)] \\
&= \left(EU^3\right)\left(EW^3\right) - 3\left(EU^2\right)\left(EW^2\right)(EU)(EW) + 3(EU)^3(EW)^3 - (EU)^3(EW)^3 \\
&= \left(EU^3\right)\left(EW^3\right) - 3\left(EU^2\right)\left(EW^2\right)(EU)(EW) + 2(EU)^3(EW)^3 \\
&= \left(\mu_{ik}^3 + 3\mu_{ik}\right)\left(\nu_{ik}^3 + O(N^{-1})\right) \\
&\quad - 3\left(1+\mu_{ik}^2\right)\left(\nu_{ik}^2 + O(N^{-1})\right)\cdot\mu_{ik}\cdot\left(\nu_{ik} + O(N^{-1})\right) \\
&\quad + 2\mu_{ik}^3\left(\nu_{ik} + O(N^{-1})\right)^3 \\
&= O(N^{-1}).
\end{aligned}
$$

So the skewness is

$$
\operatorname{SK}(UW) := \frac{E\left(UW - E(UW)\right)^3}{\operatorname{Var}(UW)^{3/2}} = O(N^{-1}).
$$

□

Next, we prove that both the median and mean of t_{ik} converges to $\sqrt{\frac{1}{2}}\cdot t_i^*$, up to an $O(N^{-1})$ difference.

Lemma 1.10. *The conditional median of t_{ik} is*

$$
\operatorname{Med}(t_{ik}|\epsilon_{i\cdot}) = \frac{\bar{x}_i^A - \bar{x}_i^B - d_k}{\sqrt{\sigma_k^2 + \hat{\sigma}_{\epsilon,i}^2}\cdot\sqrt{\frac{1}{N_A} + \frac{1}{N_B}}} + O(N^{-1}). \tag{18}
$$

In particular, for $k \in \mathcal{S}^0$,

$$
\operatorname{Med}(t_{ik}|\epsilon_{i\cdot}) = \sqrt{\frac{1}{1 + \frac{(\hat{\sigma}^p_{\epsilon,i})^2}{\sigma_k^2}}}\cdot t_i^* + O(N^{-1}). \tag{19}
$$

For $k \in \mathcal{S}^1$

$$
\operatorname{Med}(t_{ik}|\epsilon_{i\cdot}) = O(N). \tag{20}
$$

Proof. Based on Cornish-Fisher expansion,

$$\mathrm{Med}(t_{ik}|\epsilon_{i\cdot}) = E(t_{ik}|\epsilon_{i\cdot}) + \frac{\mathrm{SK}(t_{ik}) \cdot (E(t_{ik}|\epsilon_{i\cdot}) - 1)}{6\sqrt{N}} + O(N^{-1}). \tag{21}$$

However, the skewness of t_{ik} is of order N^{-1}. so

$$\mathrm{Med}(t_{ik}|\epsilon_{i\cdot}) = E(t_{ik}|\epsilon_{i\cdot}) + O(N^{-1}) = \frac{\bar{x}_i^A - \bar{x}_i^B - d_k}{\sqrt{\sigma_k^2 + \hat{\sigma}_{\epsilon,i}^2} \cdot \sqrt{\frac{1}{N_A} + \frac{1}{N_B}}} + O(N^{-1}).$$

□

Putting all the above conclusions together, we have the following theorem.

Theorem 1.11. *Assume that $\sigma_k^2 \equiv \sigma^2$ for all k (interchangeable covariance structure). The conditional mean and median of t_{ik}, for $k \in \mathcal{S}^0$, have the following asymptotic representation.*

$$\begin{aligned} E(t_{ik}|\epsilon_{i\cdot}) &\xrightarrow{\mathcal{P}_{\epsilon_{i\cdot}}} \sqrt{\frac{1}{2}} \cdot t_i^* + O(N^{-1}), \\ \mathrm{Med}(t_{ik}|\epsilon_{i\cdot}) &\xrightarrow{\mathcal{P}_{\epsilon_{i\cdot}}} \sqrt{\frac{1}{2}} \cdot t_i^* + O(N^{-1}). \end{aligned} \tag{22}$$

Here $\mathcal{P}_{\epsilon_{i\cdot}}$ is the probability law of $\epsilon_{i\cdot}$. Furthermore, for $k \in \mathcal{S}^1$, both the mean and median approaches infinity with rate $O(N)$.

Proof. We only need to use the strong law of large number (**SLLN**) of $\mathcal{P}_{\epsilon_{i\cdot}}$,

$$\hat{\sigma}_{\epsilon,i}^2 \xrightarrow{\mathcal{P}_{\epsilon_{i\cdot}}} \sigma^2.$$

Therefore

$$\sqrt{\frac{1}{1 + \frac{(\hat{\sigma}_{\epsilon,i}^p)^2}{\sigma_k^2}}} \xrightarrow{\mathcal{P}_{\epsilon_{i\cdot}}} \sqrt{\frac{1}{2}}.$$

The divergence result for $k \in \mathcal{S}^1$ follows from the fact that $\frac{d_k}{\sqrt{\sigma_k^2 + \hat{\sigma}_{\epsilon,i}^2} \cdot \sqrt{\frac{1}{N_A} + \frac{1}{N_B}}}$ is the dominating term if $d_k \neq 0$. □

Remark. Equations (22) justifies the use of multiplicative coefficient $\sqrt{2}$ in `super-delta` method when estimating the oracle t-statistic.

The divergence result for t_{ik}, $k \in \mathcal{S}^1$, implies that these statistics will "move away" from the bulk distribution that converges to a (constant times) standard Student t-distribution. This phenomenon is illustrated in Figure 1.

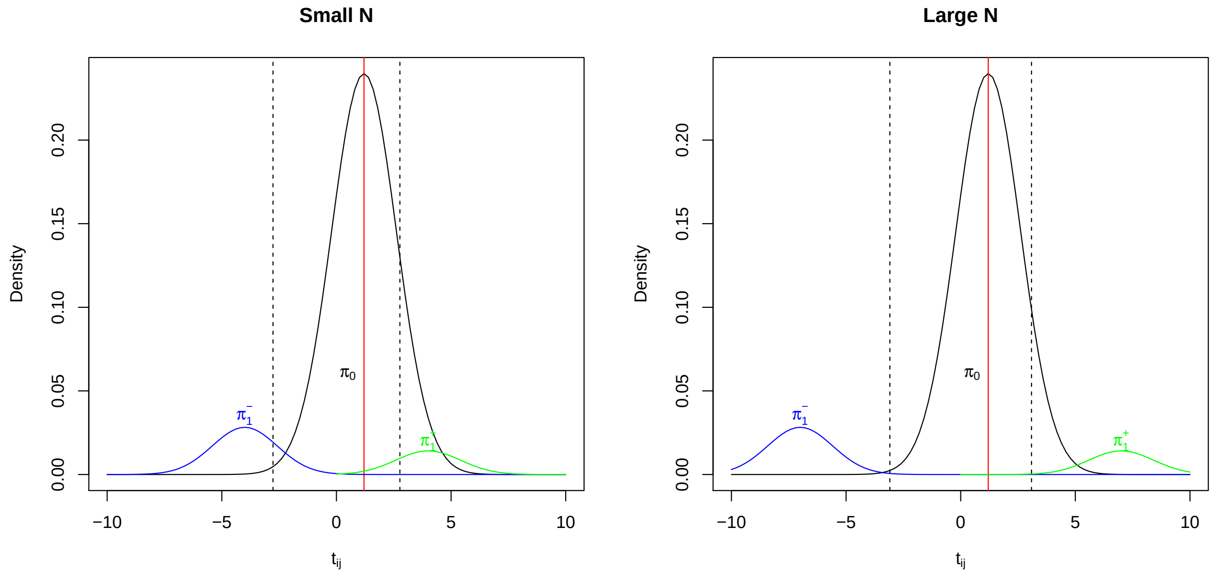

Figure 1: An illustration of different asymptotic behavior of t_{ik}. The large central density represents those t_{ik} paired with NDEGs ($k \in \mathcal{S}^0$). Two smaller densities represents t_{ik} calculated from pairing with up- and down-regulated genes. The proportions are: $\pi_0 = 0.85$ for true NDEGs; $\pi_1^+ = 0.05$ for down-regulated genes; $\pi_1^- = 0.10$ for up-regulated genes. Oracle t^* is set to be 1.7 (represented by the red vertical line). When the up- and down-regulation effect sizes are fixed and N becomes larger, the centers for the two smaller densities of t_{ik} converges to $-\infty$ and $+\infty$, respectively. Consequently, a 20% median fold trim (represented by the two dotted vertical lines) can effectively remove the two smaller clusters of t_{ik}.
